# A Novel Application of Virus Like Particles in the Hemagglutination Inhibition Assay

**DOI:** 10.3390/ijms25168746

**Published:** 2024-08-11

**Authors:** Mohamed H. El-Husseiny, Peter Pushko, Irina Tretyakova, Naglaa M. Hagag, Sara Abdel-Mawgod, Ahmed Shabaan, Neveen R. Bakry, Abdel Satar Arafa

**Affiliations:** 1 Reference Laboratory for Veterinary Quality Control on Poultry Production (RLQP), Animal Health Research Institute (AHRI), Agriculture Research Center (ARC), Giza 12618, Egypt; naglaahagagahri@gmail.com (N.M.H.); drsara_vet2006@yahoo.com (S.A.-M.); ahmedshabaansaad@gmail.com (A.S.); nevo_talk@yahoo.com (N.R.B.); abd.arafa@gmail.com (A.S.A.); 2 Medigen, Inc., Frederick, MD 21701, USA; ppushko@medigen-usa.com (P.P.); itretyakova@medigen-usa.com (I.T.)

**Keywords:** avian influenza, hemagglutination inhibition assay, virus like particles, vaccinal seed

## Abstract

The hemagglutination inhibition (HI) assay is a traditional laboratory procedure for detection and quantitation of serum antibodies of hemagglutinating viruses containing the hemagglutinin (HA) gene. The current study aimed to investigate the novel use of virus like particles (VLP) as an antigen for the HI assay. VLPs were prepared from a strain of H5N1 using a baculovirus expression system. The VLPs were characterized using the hemagglutination test, Sodium dodecyl-sulfate polyacrylamide gel electrophoresis (SDS-PAGE), Western blotting, and transmission electron microscopy. The comparative HI assay was performed using three different seed antigens: A/chicken/Mexico/232/94 (H5N2), A/chicken/Egypt/18-H/09(H5N1) and A/goose/Guangdong/1/1996(H5N1). The HI assay of serum antibody titrations using homologous antigens to these vaccinal seeds were compared to the VLP’s antigens for the same serum. The HI titers were logically relevant to the similarity between VLP antigens and vaccinal seeds, indicating the VLPs behave similarly to the standard HI assay which uses inactivated whole virus as an antigen. VLPs could be considered as an alternative to the HI assay antigen as they show a relatedness between the similarity with vaccinal seed and serum antibodies. Compared to typical entire H5N1 viral antigen prepared in SPF eggs that require proper inactivation to avoid any public health risk, VLPs prepared in tissue culture, plants or insect cells are a safe, inexpensive and scalable alternative to inactivated whole virus antigen.

## 1. Introduction

Avian influenza virus (AIV) causes outbreaks in different bird species [1]. The high economic losses due to the highly pathogenic avian influenza viruses has attracted present global attention as the mortality rate may reach 100% in poultry within a few hours. Moreover, there has been an adverse impact of certain subtypes on public health [2]. The genome of influenza viruses is segmented, consisting of eight single-stranded, negative sense RNA molecules *(PB2, PB1, PA, HA, NP, NA, M, NS)*, which encode 12 proteins; PB1, PB2, PA, NP, HA, NA, M1, M2, NS1, NEP/NS2, PB1-F2 and PB1-N40 [3,4,5]. Other than its function in receptor-binding and fusion, the hemagglutinin (HA) glycoprotein is the main surface antigen on the influenza virus. The HA protein is a major antigen which can be recognized by the adaptive immune system of the host. Neutralizing antibodies can cause selection pressure that can result in escape mutants. These mutants are typically identifiedin the HA1 domain. The accumulation of gradual changes in the antigenic structure of HA1 in viruses that circulate is referred to as antigenic drift which triggers the production of new vaccines that match the circulating strains [6,7]. The HA protein can bind to N-acetylneuraminic acid-containing proteins that are present on avian and mammalian erythrocytes, resulting in an agglutination reaction forming a diffuse lattice preventing erythrocytes from settling out or precipitating [8]. Agglutination of erythrocytes is the basis of the hemagglutination assay, while the hemagglutination inhibition (HI) assay is based on the inhibition of the agglutination response by HA subtype-specific antisera [9]. The HI assay is a traditional laboratory procedure for the classification or subtyping of hemagglutinating viruses, and moreover, the detection and quantitation of serum antibodies to these viruses such as the avian influenza viruses. Briefly, viral antigen is incubated with a dilution of serum. The result is calculated by the highest dilution of serum that inhibits hemagglutination [10,11]. Usually, antigens for the HA and HI assays are prepared by inactivating influenza virus grown in specific antibody negative or specific pathogen free (SPF) eggs using formalin or beta-propiolactone [12] that add more cost for the preparation of the antigen. In the case of highly pathogenic H5N1 viruses, this requires special precautions such as biosafety level-3+ (BSL-3+) to ensure safety, which restricts work on HPAI viruses [13]. Moreover, the inactivating agents alter viral components such as the HA proteins, which are responsible for antigenicity and immunogenicity [14], and so could affect the binding of serum with antigen and give an inappropriate HI titer. To achieve a balance between efficacy, simplicity and affordability that is urgently required in veterinary fields, the choice of VLPs as an antigen for the HI assay can have advantages in comparison to conventional inactivated whole virus antigen. Influenza VLPs are a new generation of egg-independent candidate antigens based on in vitro expression of influenza genes that encode three influenza virus proteins, hemagglutinin (HA), neuraminidase (NA), and matrix (M1) [15]. The current unprecedented study aimed to investigate the feasibility of using virus like particles as an alternative antigen source for the HI assay.

## 2. Results

### 2.1. Gene Sequence Optimization and Generation of Recombinant Baculoviruses for H5N1 VLP Expression

**Figure 1:**
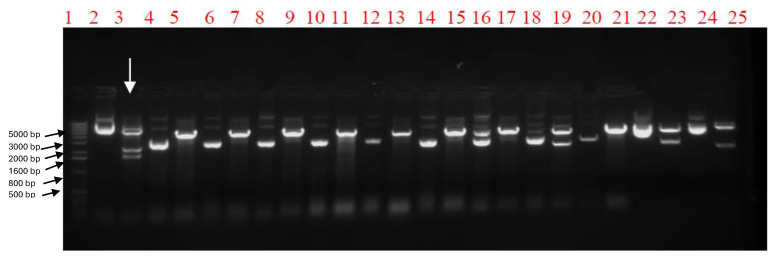
Gel electrophoresis of the restriction enzyme digestion screening of the combined pFastBac1 vector containing the three genes (*HA, NA and M1*). Lane 1 indicates the 1 Kilo base (kB) ladder (500 bp, 800 bp, 1600 bp, 2000 bp, 3000 bp, 4000 bp, 5000 bp, etc.). The even No. lanes are combined pFastBac1 vectors that are undigested by restriction enzyme Tth111I and are considered the control, while the odd No. lanes are combined pFastBac1 vectors digested by restriction enzyme Tth111I. The figure shows that the colony in lane No. 3 (marked by the white arrow) is the only one that gave specific bands (5158 bp, 2211 bp, 1719 bp) with the negative control of the same colony without restriction enzyme digestion as shown in lane 2.

**Figure 2:**
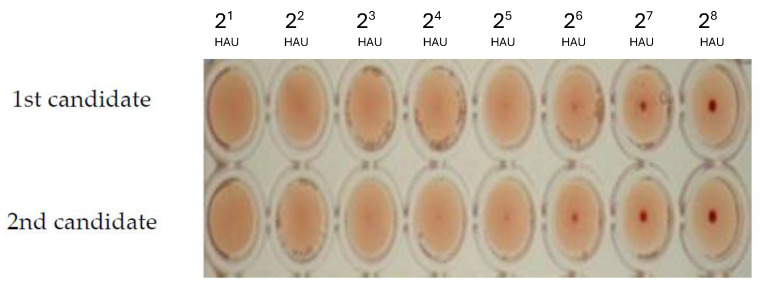
The hemagglutination assay (HA assay) of the recombinant baculovirus yielded from passage 1 (P1) using 1% of RBCS. The figure shows the HA activity of the passage 1 harvest at 2^6^ hemagglutination unit (HAU) titers, approximately. The two rows show HA titer for two different recombinant baculovirus candidates.

The nucleotide identity percentage between the A/chicken/Egypt/121/2012 (H5N1) VLP viral seed sequence and the optimized sequences for the insect cell expression of the *HA, NA* and *M1* genes were 75%, 74% and 74%. At the same time, the amino acid identity percentage between the VLP viral seed sequence and the optimized sequence for the insect cell expression of the three genes was 100%. The combined pFastBac1 vector containing the indicated genes was detected by restriction enzyme digestion screening using the Tth111I restriction enzyme. A specific colony would provide three DNA bands at specific molecular weights of 5158 base pair (bp), 2211 bp, and 1719 bp as shown in Figure 1. The recombinant baculovirus was rescued after transfection of recombinant Bacmid into Spodoptera frugiperda (SF9) insect cells. The two candidate recombinant baculoviruses were confirmed by the HA assay as shown in Figure 2, with a 2^6^ titer of HA activity. The hemagglutination activity emphasizes the proper expression of the HA protein, which is functional and hemagglutinates the blood. The HA test here, is considered the basis of selection of the proper recombinant baculovirus carrying the hemagglutinating protein.

### 2.2. VLP Production and Characterization

**Figure 3:**
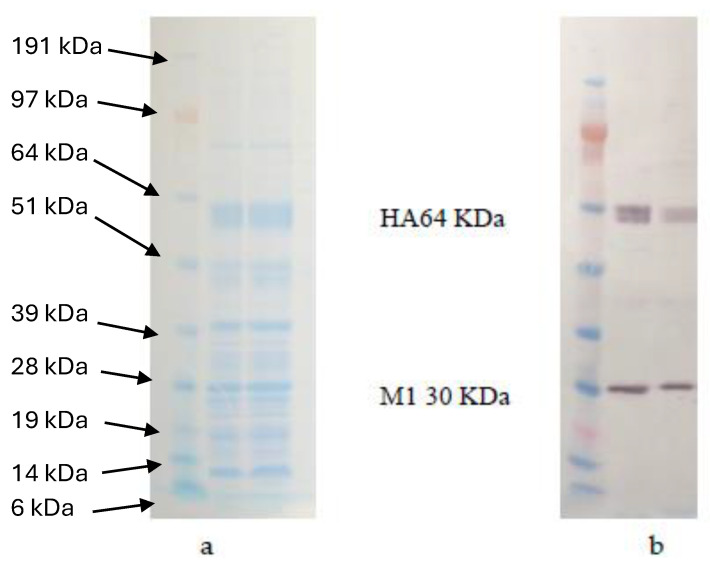
Characterization of VLP-expressed proteins using SDS-PAGE and Western blot. Figure (**a**) shows the VLP proteins separated using 4–12% gradient SDS-PAGE. The protein ladder used is SeeBlue^®^ Plus2 Pre-Stained Standard (Invitrogen). The gel shows the full-length HA protein (64 kDa) and M1 protein (30 kDa). NA protein did not appear as it was expressed less than the other influenza virus proteins. Figure (**b**) shows the Western blot of the specific band of the whole HA and M1.

**Figure 4:**
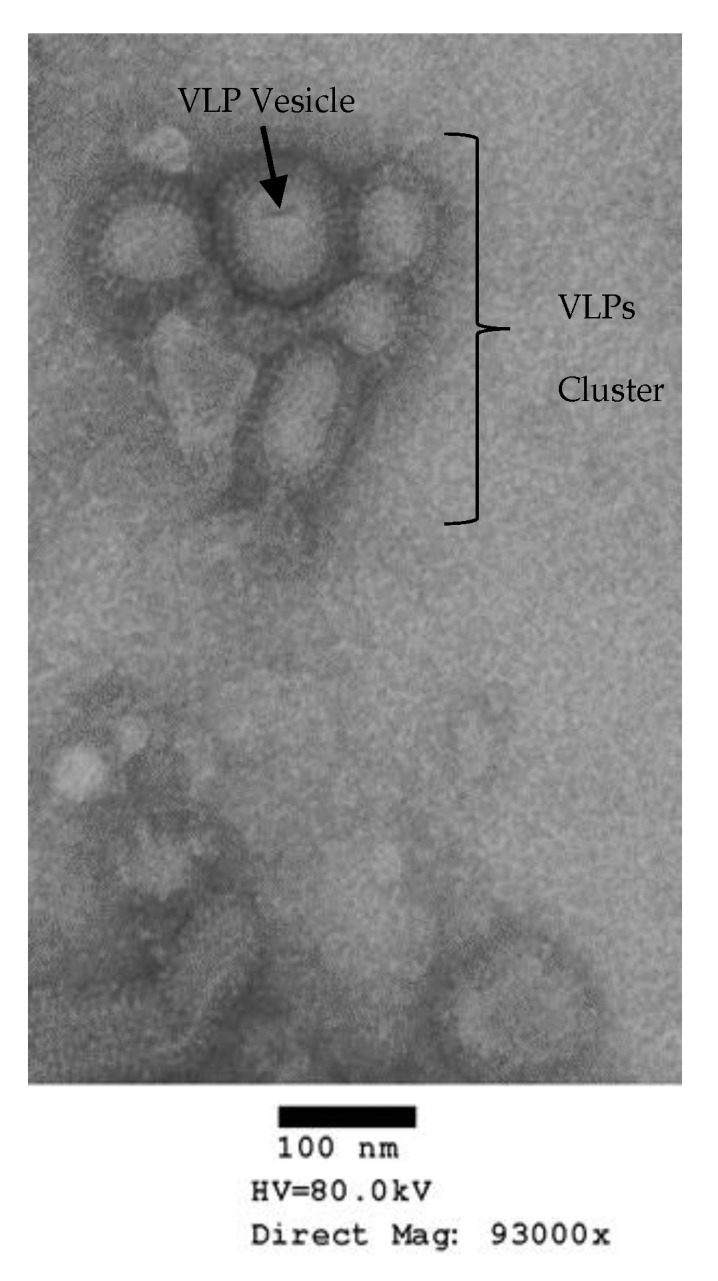
Characterization of VLPs using negative stain transmission electron microscopy. The micrograph shows the spherical VLPs with the spikes of HA and NA proteins protruding from its envelope and resembling the natural virus particles. The size bar indicates the size of the VLP particles. The normal size varies from 80 to 120 nm, the natural Avian Influenza virus size.

The VLPs were produced by passaging the confirmed recombinant baculoviruses in SF9 cells by using a multiplicity of infection (MOI) of 3. The expressed proteins were detected by using 4–12% PAGE which shows the corresponding bands of HA protein ~64 Kilo Dalton (kDa) and for M1 ~28 kDa, as shown in Figure 3a. VLP proteins (HA and M1) were confirmed by Western blotting as shown in Figure 3b, using a polyclonal antiserum against avian influenza H5 subtype. Electron micrographs detected the spherical VLPs with a diameter of nearly 120 nm, consisting of an M1 protein core and a lipid envelope containing the HA and NA proteins (Figure 4).

### 2.3. Hemagglutination Inhibition (HI) Assay and Mutation Analysis Results

**Figure 5:**
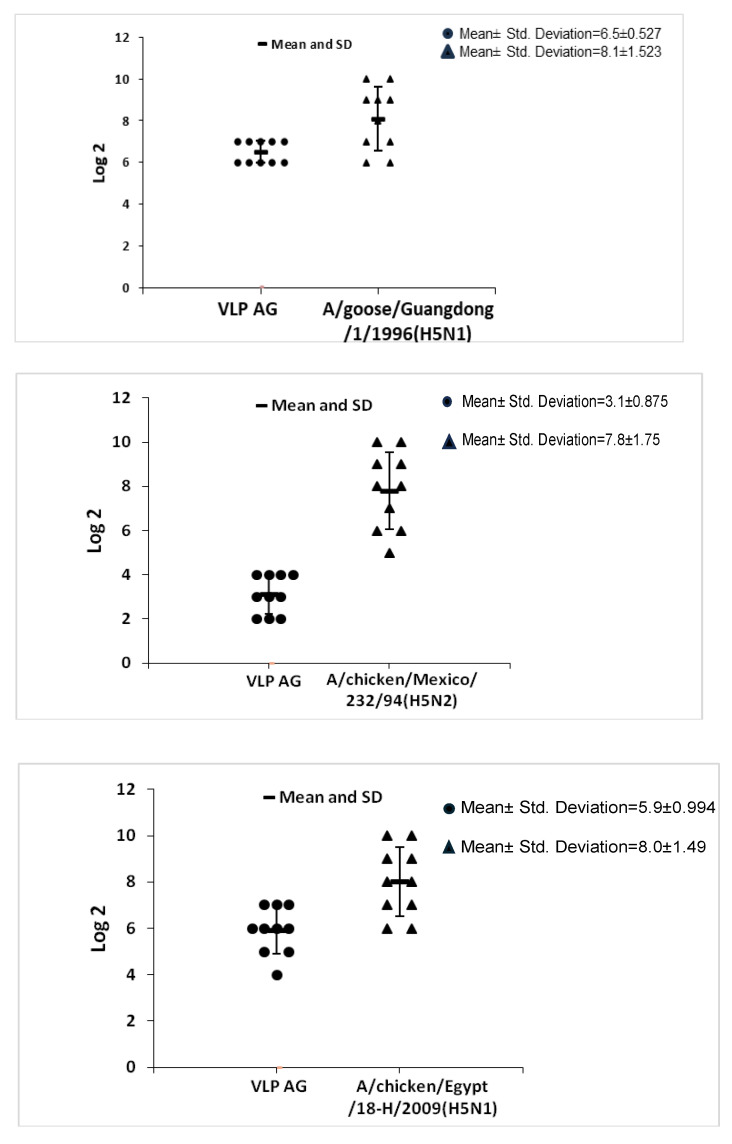
Individual HI titers of the different serum antibody groups using homologous relevant vaccinal antigens (the antibodies derived from chickens vaccinated by the same antigen used in the HI assay) against the heterologous VLP antigen.

**Figure 6:**
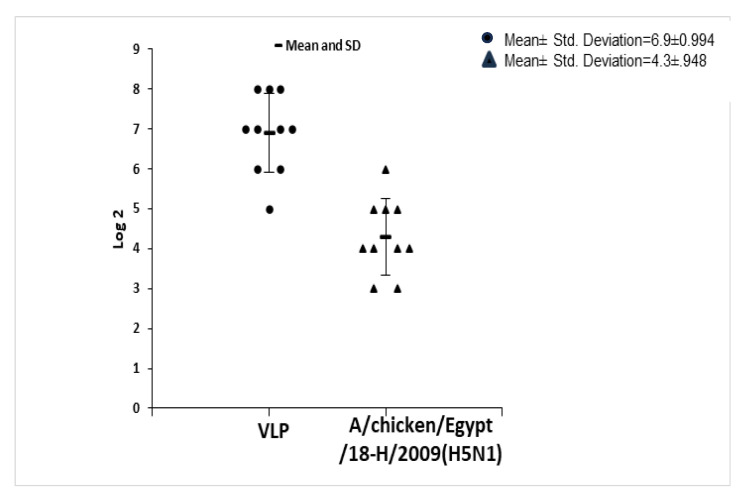
Individual HI titer of serum antibodies from SPF chickens vaccinated by VLPs using the homologous relevant vaccinal antigen (VLP) against heterologous the A/chicken/Egypt/18-H/2009(H5N1) antigen.

**Figure 7:**
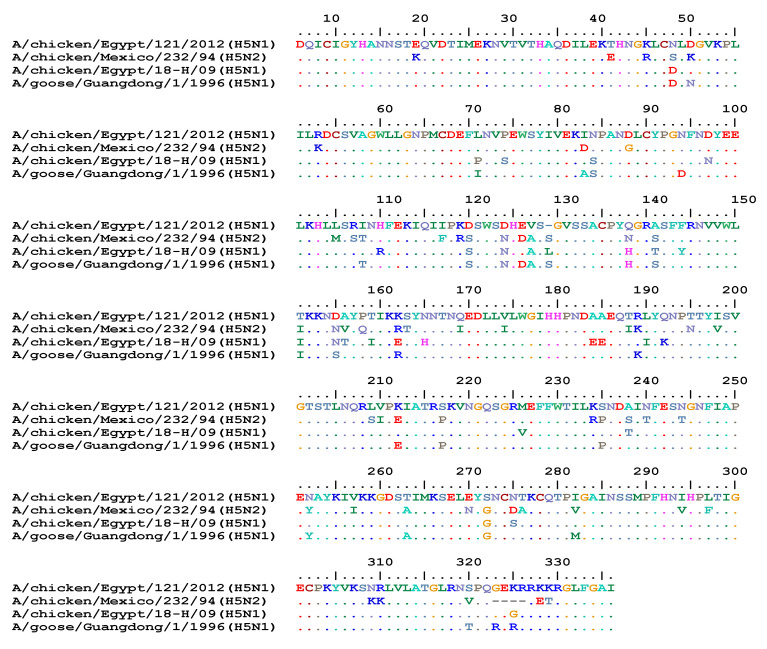
Alignment of the H5 HA1 amino acid sequences (excluding signal peptide) of the different vaccinal seeds used in this study, including the prepared VLPs’ seed. The HA1 includes the residues relevant to the epitopes in the predetermined major antigenic sites A–D. Single letter codes are shown for amino acids and colors indicate their major biochemical properties such as Red-acidic; Blue-basic.

The mean titers of the HI assay for each group of serum, using both relevant vaccinal seed and VLPs as an antigen, are addressed in Table 1 and the individual titers are shown in Figure 5 and Figure 6. Amino acid alignment shows different mutations in the HA1 protein including the important antigenic sites as clarified in both Figure 7 and Table 2. These findings showed the most matches in the amino acid sequence of the hemagglutinin protein (13 amino acid difference in antigenic sites) were between the VLP seed (A/chicken/Egypt/121/2012(H5N1) and the A/goose/Guangdong/1/1996 (H5N1). So, the HI mean titer difference between them was the lowest value, 2^1.6^. The least matches in amino acid sequence of the hemagglutinin protein (18 amino acid difference in antigenic sites) was between the VLP seed (A/chicken/Egypt/121/2012(H5N1) and the A/chicken/Mexico/232/94 (H5N2). So, the HI mean titer difference between them was the highest value, 2^4.7^.

## 3. Discussion

The highly pathogenic avian influenza virus is an important viral pathogen to the World Organization of Animal Health as it causes a devastating disease in poultry. Consequently, it has a negative impact on the global trade of poultry products [8]. Vaccination is becoming the standard tool for minimizing the economic losses in the poultry industry and maximizing food security in developing countries. Inactivated whole virus, oil-emulsified vaccines are widely used vaccines. When administered correctly, these vaccines can generate a high level of antibodies against the HA protein, resulting in a reduction in viral shedding which helps to control the disease [17]. There is a significant correlation between HA antibody titer and protection against influenza infection. A bird will be protected from death if its HI titer is over 40 HIU, while HI titers of more than 120 HIU stop virus replication depending on the antigenic match between the vaccine seed and field strains [18,19]. Screening of neutralizing antibodies against AI viruses is required to understand the vaccine-induced immunity that is essential for control measures of the disease. Several immunological techniques can be used to quantify virus specific antibody titers. These include solid-phase Enzyme-linked immunosorbent assay (ELISA), bead-based ELISA, serum neutralizing assays and the HI assay [8,20,21,22]. Because of its simplicity, the HI assay has been used for a long time to screen virus-neutralizing antibodies against HA proteins in serum [23]. The current study is aimed at assessing VLPs as an alternative to the inactivated virus antigen commonly used in the HI assay. To the best of our knowledge, this has not been studied previously. The VLPs were always used as a vaccine candidate or a delivery platform [24,25,26]. The results of this study illustrated that VLPs can be successfully used as an antigen in the HI assay. The VLPs of H5N1 clade 2.2.1.2 virus origin were tested as an antigen in the HI assay for screening neutralizing antibodies to vaccinal seeds of different clades: such as A/goose/Guangdong/1/1996 (H5N1) (Re1 seed) clade 0, A/chicken/Egypt/18-H/2009 (H5N1) clade 2.2.1.1 from the Egyptian strain and finally, A/chicken/Mexico/232/94 (H5N2), a classic reference vaccine seed [27]. The HI titers using VLP as an antigen were logically relevant to the amino acid matches between VLP antigen seeds and vaccinal seed sequences, indicating the VLPs behave similar to the standard HI assay, which uses inactivated whole virus as an antigen. The most dissimilar vaccine seed to the VLP antigen seed in this study was the A/chicken/Mexico/232/94 (H5N2). Antibody titers derived from this vaccine showed a difference about 2^4.7^ HIU between VLPs as a heterologous HI antigen and homologous vaccinal HI assay antigen as shown in Table 1. In the same context, this dissimilar vaccine showed the highest number of mutations related to the VLP antigen. There are 18 mutations at the antigenic sites and escape mutant positions [16]. On the other hand, the most similar vaccine seed to the VLP antigen seed in this study was the A/goose/Guangdong/1/1996 (H5N1) (Re1 seed). The HI titer read a minimal difference between the homologous antigen and the heterologous VLP antigen of about 2^1.6^ HIU as shown in Table 1. Also, the least number of mutations were observed between this vaccine virus and the VLPs. The last vaccine virus, A/chicken/Egypt/18-H/2009(H5N1), was used to compare with the VLPs’ antigen. It has 17 mutations in the antigenic sites and escape mutant positions. The difference between the HI read of the two antigens was 2^2.1^ HIU. This is slightly above the difference read of 2^1.6^ HIU of the most similar antigen. In addition, this result is different to the most dissimilar antigen (2^4.7^ HIU), although the antigen has only one fewer mutation than the most dissimilar antigen (18 mutations). This may be due to biological variations such as inherent differences in the immunogenicity of the HA protein [28]. These results suggest that VLPs support the HI assay similarly to standard, traditional, inactivated whole virus. The titer of serum antibodies correlates to the relatedness between the HI assay antigen and the vaccine seed [10]. To validate this explanation, we reversed the experimental situation. Serum was collected from SPF vaccinated chickens using VLPs as vaccinal antigen and tested for antibodies by the HI assay using the homologous VLPs’ antigen and the heterologous A/chicken/Egypt/18-H/2009(H5N1) antigen. The difference between the two readings was 2^2.6^ HIU, which is similar to the difference of the HI assay reading when VLPs are the heterologous antigen and A/chicken/Egypt/18-H/2009(H5N1) is the homologous antigen and that resulted in 2^2.1^ HIU. Fortunately, Speckman and her colleagues [28], used the same two vaccinal strains to measure serum antibodies using the homologous vaccinal strain antigen in the HI assay and eight different strains as heterologous antigens in the same assay. Their study denoted that there was a wide variation in antibody levels between homologous and heterologous strains of different clades and similarities. These results support the application of VLPs as a heterologous antigen using the HI assay against different clades of vaccinal strains in accordance with their similarity.

Influenza VLPs are based on in vitro expression of certain influenza genes that encode main structural proteins, without assembly of any nucleic acid segments which are necessary for replication [12], making VLPs safer than whole inactivated virus. VLPs can be prepared from only hemagglutinin (HA) and matrix (M1) proteins without neuraminidase [29]. VLPs can be used as an antigen for the HI assay without steric inhibition caused by neuraminidase which can alter titration of serum antibodies. Also, VLPs without neuraminidase protein can be used for production of sera for influenza typing without interference by steric inhibition, which can occur when using sera prepared from whole virus antigen that causes false positive results [10].

VLPs can co-localize and display different hemagglutinin proteins from different subtypes in the same VLP construct [30], or even prepared safely in a heterosubtypic model [31]. In both cases VLPs can be used as a universal antigen for the HI assay in a fashion suitable for quantitation of serum antibodies of different influenza subtypes. This is not always possible with whole virus antigen due to safety concerns, especially for HPAI strains. Cost is also a significant issue in diagnosis and control measures, especially in the veterinary field. The cost of preparation of VLPs in insect cell tissue culture by using a baculovirus expression system or plant derived virus is lower than the cost of the cultivation of whole virus in SPF eggs and the inactivation process [32,33], suggesting that recombinant VLPs can be a cost-effective antigen for HI assays.

## 4. Materials and Methods

### 4.1. Optimization and Biochemical Synthesis of HA, NA, and M1 Genes

As previously published [34], the main genes (*H5, N1, and M1*) for construction of VLPs of the avian influenza virus were identified and sequenced from an identified isolate, A/chicken/Egypt/121/2012 (H5N1). To maximize expression in SF9 cells (ATCC, Manassas, VA, USA), the genes’ codons were optimized for insect cells and biochemical synthesis of the genes was carried out (Genescript, Piscataway, NJ, USA).

### 4.2. Generation of Recombinant Baculoviruses

Recombinant baculovirus (rBV) expressing *H5, N1, and M1* genes were constructed by using the Bac-to-Bac baculovirus expression system^®^ (Invitrogen, Carlsbad, CA, USA). Firstly, the optimized full-length *H5, N1, and M1* genes were cloned into pFastBac1 transfer vector. Afterwards, the three genes were combined within a single pFastBac1 transfer vector by using HpaI, SnaBI and PvuI restriction enzymes, as previously described [15]. Secondly, the recombinant bacmid was produced by site-specific transposition, with Tn7 used to insert the *H5, N1, and M1* genes from the combined recombinant pFastBac1 transfer vector (donor vector) into the bacmid DNA containing the AcMNPV baculovirus genome that occurs in E. coli DH10Bac^®^ competent cells (Invitrogen, Carlsbad, CA, USA) after transformation with the recombinant transfer vector. Finally, the Sf9 insect cells (Invitrogen, Carlsbad, CA, USA) were transfected by the recombinant bacmid using Fugene^®^ (Promega, Madison, WI, USA) to produce recombinant baculoviruses.

### 4.3. Protein Expression, Purification, and Characterization of VLPs

The recombinant baculovirus was titered by the plaque assay after passaging in Sf9 insect cells using SF-900 II SFM^®^ serum-free medium (Gibco, Grand Island, NY, USA). For protein expression, Sf9 cells were infected for 72 h at a cell density of 2 × 10^6^ cells/mL with recombinant baculoviruses at a MOI = 3. Culture supernatants were harvested and clarified by centrifugation at 3000 rpm/15 min at 4 °C. The supernatant was filtered using a sterile 0.2 µm filter. The filtrate was ultracentrifuged to isolate VLPs. The VLPs were characterized by SDS–PAGE using 4–12% gradient polyacrylamide gels (Invitrogen, Carlsbad, CA, USA) and by Western blotting using specific sera. The characterized VLP samples were adsorbed onto grids for electron microscopy (Poly Sciences, Warrington, PA, USA). The grids were negatively stained with 1% phosphotungstic acid. Then, the grids were visualized on a Hitachi H-7600 transmission electron microscope (Hitachi High Technologies America, Schaumburg, IL, USA).

### 4.4. Preparation of Anti-VLP Serum

Firstly, two groups of ten 7-day old chickens were used. Each group was separated in a BSL3 isolator. The first group was vaccinated twice subcutaneously (S/C) in the neck fold at 7 days and 28 days old with the VLP preparation without adjuvant. The second group was considered the negative control group and injected only with phosphate-buffered saline (PBS) in place of the VLPs. The animal experiments were conducted in the animal facility unit at Animal Health Research Institute (AHRI) and the protocol was approved by the Review Board of the Animal Health Research Institute (AHRI-2022928). The serum from the VLP-immunized SPF chickens was used to evaluate the antibody titer using the HI assay.

### 4.5. Evaluation of the VLPs as a Homologous Antigen Using the HI Assay

The HA and HI assays were performed using standard protocols [35]. Briefly, the HA activity of purified H5N1 VLPs was tested against red blood cells (RBCs) and HA titers were recorded. The HI assay was performed in V-bottom 96-well microtiter plates using 4 HAU of the VLPs as an antigen. The serum from the VLP-immunized SPF chickens were subjected to a two-fold serial dilution with PBS, then the VLPs were added as antigen prior to the addition of 1% chicken red blood cells. The HI titer is the highest dilution of serum that inhibits hemagglutination of 4 HAU of an antigen. The only wells that should be considered to exhibit inhibition are those in which the RBCs stream at the same rate as the control wells (containing only 0.025 mL RBCs and 0.05 mL PBS). The validity of the results was assessed against the negative and the positive control serum. On the other hand, the same test was conducted in the same ten serum samples of the VLPs vaccinated SPF chickens using the A/chicken/Egypt/18-H/09(H5N1) seed as a heterologous antigen for the HI assay.

### 4.6. Evaluation of the VLPs as a Heterologous Antigen Using the HI Assay

Another three groups of ten serum samples were obtained from commercial farms, from chickens vaccinated with different vaccinal seeds. The first group of serum was from commercial chickens vaccinated with the A/chicken/Mexico/232/94(H5N2) seed, the second group was vaccinated with the A/chicken/Egypt/18-H/09(H5N1) seed, and the third group of chickens was vaccinated with the A/goose/Guangdong/1/1996(H5N1) (Re1) seed. HI assays were carried out two times for these serum groups, as previously described, once using the prepared VLPs as a heterologous antigen and again using the relevant seed as a homologous antigen.

### 4.7. Mutation and Statistical Analysis

The nucleotide sequences of the HA gene of the VLP construct seed A/chicken/Egypt/121/2012 (H5N1) and the different vaccinal seeds used in this study were retrieved from gene bank. They were translated into the deduced amino acids and aligned using Bioedit 7.2 software (Ibis Biosciences, Carlsbad, CA, USA) [36] to demonstrate the mutated epitopes’ residues at different antigenic sites and the residues relevant to the escape mutants. Statistical analysis was performed by SPSS version 22 for Windows (IBM SPSS Statistics for Windows, Version 22.0. Armonk, NY, USA). The independent samples t-test and the Mann–Whitney U test were used to compare the HI titer results using VLPs as heterologous antigen with the homologous antigen, and vice versa. The statistical tests were performed using *p* < 0.05. A jittered dot plot was used to visualize the individual titers for each group.

## 5. Conclusions

In conclusion, influenza VLPs can be considered as an alternative antigen for HI assays that traditionally use whole virus as antigen. HI titers observed using VLPs as an antigen show similarity to HI titers observed with traditional vaccinal seed antigens. When similarity to vaccinal seed is high, the VLPs as an HI antigen, show titers of antibodies that are close to the titer observed using the homologous traditional HI antigen. When VLPs show less similarity to the vaccinal seed they produce an HI titer of antibodies that differs from the titer observed by using the homologous traditional HI antigen.

## Figures and Tables

**Table 1:** The mean titers of the Hemagglutination Inhibition (HI) assay and the amino acid identity between the vaccine seed and the antigen used in the HI assay for the different groups.

No.	Vaccine Seed	Accession No.	HI Antigen	HI Mean Titer, log2 ± Std. Deviation	*p* Value	Identity% between Vaccine Seed and HI Antigen	* The HI Mean Titer Difference between Homologous and Heterologous Antigen
1	A/chicken/Egypt/18H/2009(H5N1)	CY062601.1	A/chicken/Egypt/18-H/2009(H5N1)	2^8^ ±1.49	0.002	100%	2^2.1^
			A/chicken/Egypt/121/2012(H5N1) (VLP seed)	2^5.9^ ±0.994		92%	
2	A/goose/Guangdong/1/1996(H5N) (Re1 seed)	NC_007362.1	A/goose/Guangdong/1/1996(H5N1) (Re1 seed)	2^8.1^ ±1.523	0.018	100%	2^1.6^
			A/chicken/Egypt/121/2012(H5N1) (VLP seed)	2^6.5^ ±0.527		92%	
3	A/chicken/Mexico/232/94(H5N2)	AY497096	A/chicken/Mexico/232/94(H5N2)	2^7.8^ ±1.75	0.000	100%	2^4.7^
			A/chicken/Egypt/121/2012(H5N1) (VLP seed)	2^3.1^ ±0.875		82%	
4	A/chicken/Egypt/121/2012(H5N1) (VLP seed)	JQ858483.1	A/chicken/Egypt/121/2012(H5N1) (VLP seed)	2^6.9^ ±0.994	0.000	100%	2^2.6^
			A/chicken/Egypt/18-H/2009(H5N1)	2^4.3^ ±0.948		92%	

* The HI mean titer difference indicates the subtraction of the HI mean titer of both homologous and heterologous antigens. For example, in the first group the antibodies are derived from chickens vaccinated by the vaccine seed A/chicken/Egypt/18H/2009(H5N1). The HI titer was measured by two antigens. The first antigen was the same as the vaccine seed (homologous antigen) A/chicken/Egypt/18H/2009(H5N1) and the titer of the HI assay was 2^8^ Hemagglutination Inhibition unit (HIU) (similarity between vaccine seed and the HI antigen seed is 100%). The second antigen was the tested VLPs’ antigen (heterologous antigen) A/chicken/Egypt/121/2012(H5N1) and the titer of the HI assay was 2^5.9^ HIU (similarity between the vaccine seed and the HI antigen seed is 92%). So, the HI mean titer difference between homologous and heterologous antigen = 2^8^ − 2^5.9^ = 2^2.1^ HIU.

**Table 2:** Comparison between different vaccinal seeds used in this study and the prepared VLPs’ seed, listing the mutated epitopes’ residues in different antigenic sites and the residues relevant to the escape mutants.

Amino Acid No. *	A/Chicken/Egypt/121/2012(H5N1) (VLP)	A/Goose/Guangdong/1/1996(H5N1)	A/Chicken/Mexico/232/94(H5N2)	A/Chicken/Egypt/18-H/2009(H5N1)
40	K	K	R	K
43 C	N	D	S	D
115	Q	Q	Q	Q
120	D	S	S	S
123	S	S	S	S
124	D	N	N	N
126	E	D	D	E
129 A	DEL	S	S	L
138 A	Q	H	N	H
140 A	R	R	R	R
141 A	A	S	S	T
151 B	T	I	I	I
155 B	D	S	D	N
156 B	A	A	V	T
159	T	T	T	I
162	K	R	R	E
174	V	V	I	V
184 B	A	A	A	E
185 B	A	A	A	E
188 B	T	T	I	T
189 B	R	K	K	R
190 B	L	L	L	I
217 D	S	P	P	S
238 D	A	A	A	T
272 C	S	G	G	G
275 C	N	N	D	S
276 C	T	T	A	T
No. of mutations to VLPs		13	18	17

* The numbers of amino acids were previously determined [16]. A/B/C/D are the antigenic sites. The amino acid numbers that are not indicated by antigenic site symbols are substitution residues that may affect antigenic sites. They were identified by using the H5 antibody escape mutants.

## Data Availability

The original contributions presented in the study are included in the article, further inquiries can be directed to the corresponding author.
